# Synthesis and Metabolic Fate of 4‐Methylthiouridine in Bacterial tRNA

**DOI:** 10.1002/cbic.202000272

**Published:** 2020-06-18

**Authors:** Christoph Borek, Valentin F. Reichle, Stefanie Kellner

**Affiliations:** ^1^ Department of Chemistry Ludwig-Maximilians-Universität München Butenandtstr. 5–13 81377 Munich Germany

**Keywords:** epitranscriptome, modified nucleoside, NAIL-MS, RNA damage, tRNA

## Abstract

Ribonucleic acid (RNA) is central to many life processes and, to fulfill its function, it has a substantial chemical variety in its building blocks. Enzymatic thiolation of uridine introduces 4‐thiouridine (s^4^U) into many bacterial transfer RNAs (tRNAs), which is used as a sensor for UV radiation. A similar modified nucleoside, 2‐thiocytidine, was recently found to be sulfur‐methylated especially in bacteria exposed to antibiotics and simple methylating reagents. Herein, we report the synthesis of 4‐methylthiouridine (ms^4^U) and confirm its presence and additional formation under stress in *Escherichia coli*. We used the synthetic ms^4^U for isotope dilution mass spectrometry and compared its abundance to other reported tRNA damage products. In addition, we applied sophisticated stable‐isotope pulse chase studies (NAIL‐MS) and showed its AlkB‐independent removal *in vivo*. Our findings reveal the complex nature of bacterial RNA damage repair.

## Graphical abstract

**Through a combination** of synthetic and analytical chemistry, the structure of the modified nucleoside 4‐methylthiouridine was confirmed in bacterial tRNA. The modification is of low abundance and forms after bacterial stress induced by methylating agents; this argues for its nature as a natural tRNA lesion. By pulse chase analysis, we show its repair through a dethiomethylation mechanism *in vivo*.

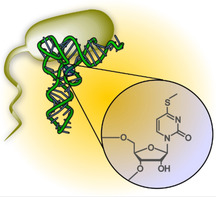

RNA and especially tRNA have complex structures to fulfill their important functions inside the organism. This is possible through the vast chemical variety of building blocks found in RNA. To date over 170 modifications to either ribose or nucleobase have been reported.^[1]^ One group of unique tRNA modifications is enzymatic thiolation. In bacteria, thiolation of uridine (4‐thiouridine, s^4^U) is commonly found at position 8 of most tRNAs (red in Figure 1). s^4^U is a target of ultraviolet light;^[2]^ it leads to a reduced growth of bacteria exposed to UV and, as a consequence, saves bacteria from photomutagenic effects.^[3]^ In addition, s^4^U‐hypomodified tRNAs were found to be targeted by the RNA degradosome; this leads to a reduced abundance of a subset of bacterial tRNAs.^[4]^ Due to its sulfur decoration, s^4^U is a nucleophile, and can be coupled with electrophiles such as bromomethylcoumarin^[5]^ or iodoacetamide.^[6]^ The latter is used to assess RNA transcription and stability after metabolic RNA labeling with exogenous s^4^U (SLAM‐Seq). Similar to SLAM‐Seq, TUC‐Seq uses metabolically introduced s^4^U, which can be chemically converted to cytidine prior to RNA sequencing^[7]^. Despite its important function in bacterial tRNA and its broad use as a metabolic label for RNA sequencing, little is known about its chemical reactivity inside cells.

**Figure 1:**
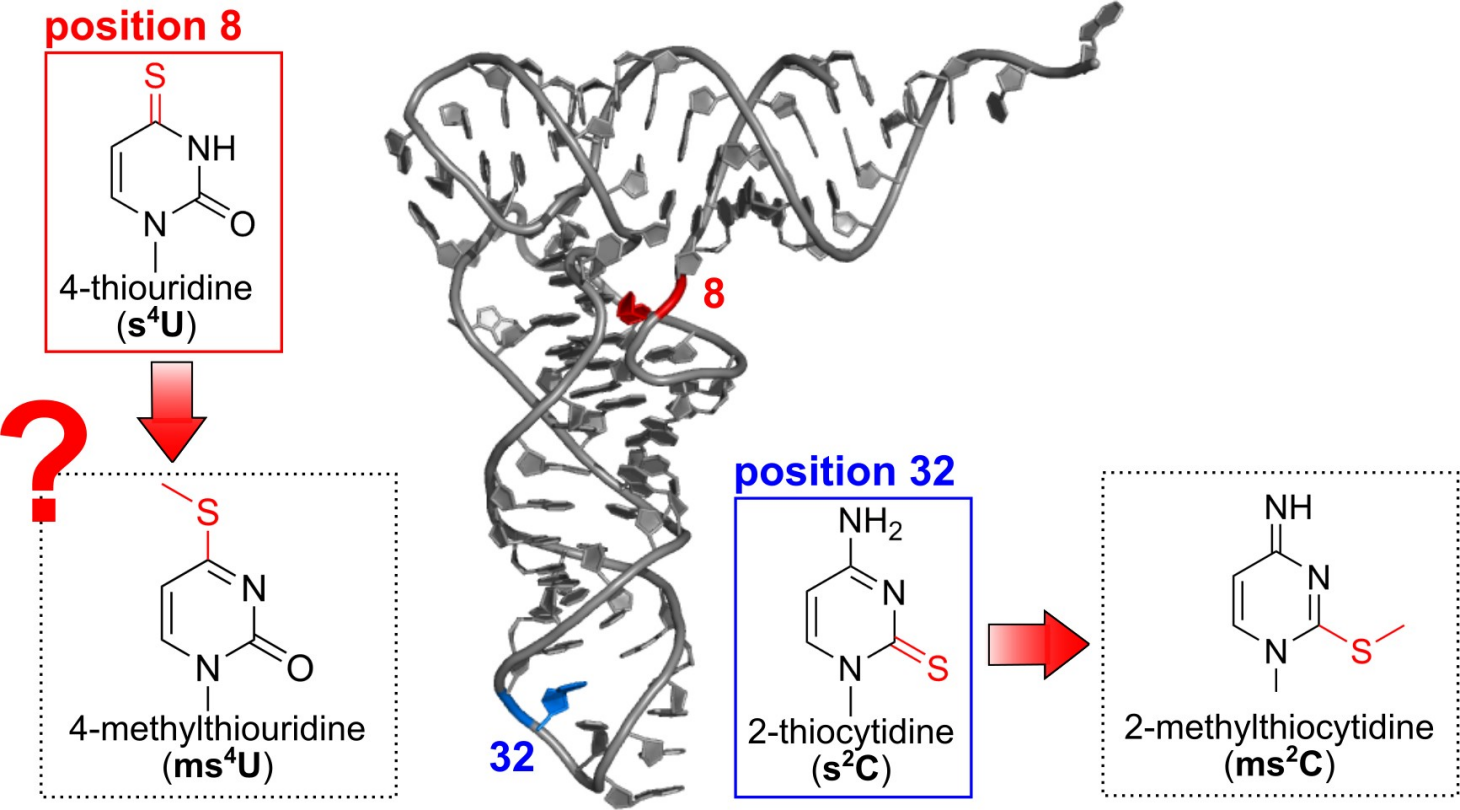
3D structure of tRNA indicating the positions of enzymatically thiolated nucleobases in bacteria. Red: 4‐thiouridine (**3**, s^4^U) found at position 8 and the suggested structure of its methylated derivative 4‐methylthiouridine (**4**, ms^4^U). Blue: 2‐thiocytidine at position 32 and its reported derivative 2‐methylthiocytidine.

Another sulfur decorated tRNA modification, 2‐thiocytidine (s^2^C) (blue in Figure 1) has been recently found to be endogenously methylated^[8]^ and efficiently repaired, potentially through its function as a modulator of translation.^[9]^ A direct methylation of s^2^C through electrophiles such as *S*‐adenosylmethionine, methyl methanesulfonate (MMS) or antibiotics (streptozotocin) was observed. The resulting damage ms^2^C (Figure 1) is substrate to the α‐ketoglutarate dependent dioxygenase AlkB and repaired both *in vitro* and *in vivo* to restore tRNA function.^[8]^ While s^2^C is fully accessible to electrophiles in the anticodon loop of tRNA, s^4^U is in tight interaction with nucleosides of the D‐ and T‐loop and might be less accessible to electrophiles. This raises the question of whether s^4^U is a target to direct methylation and if so, how much damage forms and how bacteria react to the damage.

To address these questions, we report here the synthesis of the suggested damage product ms^4^U (**4**).

The synthesis of ms^4^U (**4**) was first attempted *via* the formation of the fully acetylated corresponding 4‐triazolic precursor which was meant to react with sodium thiomethanolate^[10]^ to form the desired nucleoside. We encountered several problems in the key step due to partial deprotection of the ribose moiety, which led to further problems with the purification. Therefore, we decided to form ribose‐protected 4‐thiouridine (**2**) separately with subsequent methylation adopting a procedure for the corresponding 2′‐chlorine riboside.^[11]^ The complete reaction is shown in Scheme 1. The initial peracetylation in neat acetic anhydride with catalytic amounts of iodine is a fast and reliable method to protect sugars in general which provided conversion of uridine to compound (**1**) in high yields. The subsequent formation of the 4‐thiouridinic compound (**2**) by thiolation with phosphorus pentasulfide yielded 72 %. It should be noted, that crystallization from ethanol, as described for the chlorinated compound, could not be observed. The deprotection was conducted by refluxing in concentrated aqueous ammonia solution, and s^4^U (**3**) was received presumably in quantitative yield but was used as crude product in the next step. Of note, the more common method under Zemplén conditions^[12]^ was not capable of deprotecting compound (**2**). In a final step, the thio group was selectively methylated by iodomethane to provide ms^4^U (**4**) in a moderate overall yield of 40 % over four steps.

**Scheme 1:**
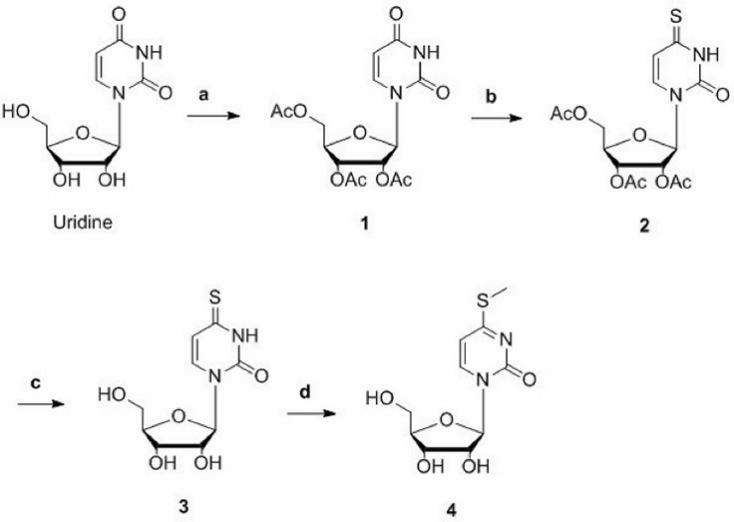
Synthesis of the key compound **4**: a) Ac_2_O, I_2_, RT, 45 min; b) P_2_S_5_, pyridine, reflux, 4 h; c) NH_4_OH conc., reflux, 2 h; d) MeI, EtOH 50 %, RT, 1 h.

With the synthetic standard in hands, we developed a sensitive LC‐MS/MS method for detection of ms^4^U in tRNA from unstressed *Escherichia coli*. With this targeted analysis, we found a peak in native tRNA that corresponds to the synthetic ms^4^U in terms of retention time, precursor and product ion mass. In *E. coli* exposed to the LD_50_ dose of methyl methanesulfonate (MMS), the peak increased. A co‐injection of the synthesized ms^4^U standard and tRNA from MMS exposed *E. coli* grown in stable isotope labeled medium clearly showed 1) perfect co‐elution and 2) the expected numbers of carbon, nitrogen, and sulfur atoms in native ms^4^U (Figure 2A). In a next step, we confirmed the origin of the methyl group attached to the sulfur following our established methylome discrimination assay.^[13]^ For this purpose, we grew *E. coli* in medium supplemented with [CD_3_]‐S‐methionine; this leads to CD_3_ labeling of all enzymatically placed methyl groups. After exposing *E. coli* to MMS, we found a high intensity signal for CH_3_‐methylated ms^4^U and only a minor signal for CD_3_‐methylated ms^4^U (Figure 2B). We thus prove the direct methylation of s^4^U through the electrophile MMS in bacterial tRNA *in* *vivo*.

**Figure 2:**
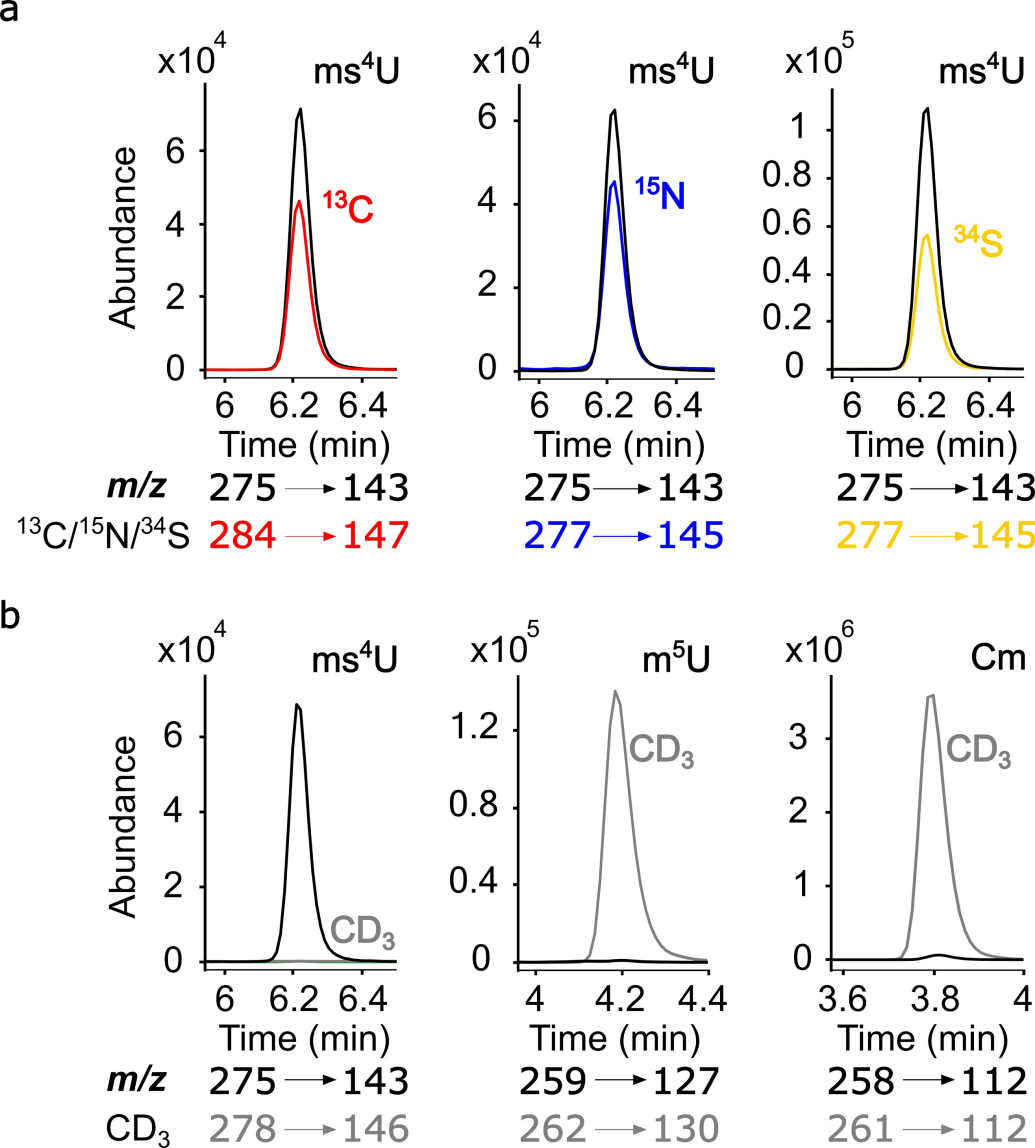
LC‐MS/MS analysis of native and synthesized ms^4^U. A) Co‐injection of synthesized ms^4^U (black) and digested tRNA from ^13^C (red), ^15^N (blue), and ^34^S (yellow) metabolically labeled *E. coli* cultures. B) Native tRNA digests screening for enzymatically methylated nucleosides (gray, [CD_3_]‐S‐methionine‐derived) and damage‐derived nucleoside methylation (black). Abbreviations: ms^4^U: 4‐methylthiouridine (**4**), m^5^U: 5‐methyluridine, and Cm: 2’‐*O*‐methylcytidine. The mass transitions (precursor ion→product ion) are given below the respective chromatograms.

We were next interested to quantify the extent of ms^4^U formation in unstressed and MMS‐treated tRNA. For this purpose, a stable isotope labeled internal standard (SILIS) of ms^4^U was produced by metabolic isotope labeling of *E. coli*. To increase the yield of stable isotope labeled ms^4^U, MMS was added to the culture medium for 60 minutes, and the RNA was harvested and processed as previously described.^[14]^ The combination of synthesized ms^4^U and metabolically produced ms^4^U‐SILIS allowed accurate quantification of ms^4^U and other modified ribonucleosides in bacterial tRNA (Figure 3). For normalization, we plotted the number of modified nucleosides per 10^6^ canonical ribonucleosides (rN).

**Figure 3:**
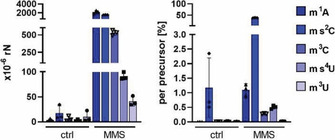
Absolute quantification of damage‐derived nucleosides found in tRNA in control *E. coli* and *E. coli* exposed to 20 mM MMS. Left: per 10^6^ rN (ribonucleosides) Right: per precursor [%]. Abbreviations: m^1^A: 1‐methyladenosine, ms^2^C: 2‐methylthiocytidine, m^3^C: 3‐methylcytidine, ms^4^U: 4‐methylthiouridine, and m^3^U: 3‐methyluridine. From 3 biological replicates. Error bars represent standard deviation.

In tRNA of unstressed *E. coli*, we found 2.6×10^−6^ ms^4^U/rN, which is less compared to the natural abundance of our recently described modification ms^2^C (17×10^−6^ ms^2^C/rN).^[8]^

After exposure to MMS, the abundance of the known tRNA damage products is 2147×10^−6^ m^1^A/rN, 1543×10^−6^ ms^2^C/rN, 2772×10^−6^ m^7^G/rN, 530×10^−6^ m^3^C/rN, 478×10^−6^ m^6^A/rN and 41×10^−6^ m^3^U/rN (Figure 3 and Figure S1a in the Supporting Information). ms^4^U damage is with 91×10^−6^ ms^4^U/rN comparable to m^3^U damage in bacterial tRNA. This value appears to be rather low, but if the abundance of damage is normalized to the abundance of its respective precursor nucleoside (*e*. *g*., m^1^A per A or ms^4^U per s^4^U) a different conclusion must be drawn. With 0.5 % ms^4^U/s^4^U, ms^4^U is of comparable abundance to the known damage product m^1^A (1.1 % per A; Figures 3 and S1b). S4 in thiouracil is thus similarly reactive towards electrophiles such as MMS as is the N1 in adenine and the N7 in guanine. However, the S2 of thiocytosine is the strongest nucleophile and thus 38 % of all s^2^C become methylated to ms^2^C in tRNA from *E. coli* exposed to MMS. Due to the importance of s^2^C during translation, where it negates the wobble inosine binding to codons starting with adenine,^[9]^ its efficient repair by enzymatic demethylation has been reported.^[8]^

s^4^U is found at position 8 in 60 %^[1]^ of all bacterial tRNAs and in addition at position 9 in tRNA^Tyr^_QUA_ from *E. coli*. The chemical properties of sulfur are exploited by the bacteria for oxidative stress sensing through, for example, UV irradiation. Oxidative stress can be triggered by UV irradiation following iron‐dependent Fenton chemistry. Therefore, s^4^U acts as a sensor for UV irradiation,^[15]^ which leads to delayed growth of bacteria during UV light exposure.^[16]^ Given this important function of s^4^U, we were wondering how cells react to tRNAs which have been methylated and carry ms^4^U. For this purpose, we designed a pulse chase study based on our NAIL‐MS expertise.

The goal of this assay is to discriminate the damaged tRNAs and exclude signals from tRNAs transcribed during recovery from MMS stress. Thus, we can follow the metabolic fate of ms^4^U/rN independently from dilution by transcription. For this purpose, cells are grown in medium containing only ^14^N and ^32^S. Consequently, the RNA is completely labeled with ^14^N, and all s^4^U have a ^32^S label (original s^4^U), for example, *m/z* (s^4^U) 261. In this medium, the bacteria are exposed to MMS (20 mM) and s^4^U is converted to ms^4^U and, for example, A to m^1^A. After exposure, MMS is removed by exchanging the medium with stable isotopes containing medium. During the following recovery period, newly transcribed tRNA will be ^15^N labeled, enzymatically methylated nucleosides will be CD_3_ labeled and new s^4^U will have a ^34^S label (new s^4^U, *m/z* 265 and new m^1^A, *m/z* 290). The experimental design is shown in Figure 4A. Using LC–MS/MS analysis, we detect the formation of ms^4^U during MMS exposure with around 50×10^−6^ ms^4^U/original rN. In the subsequent recovery period, we traced the abundance of ms^4^U and normalized it to the abundance of original rN. In wild‐type *E. coli*, we saw a constant decrease in ms^4^U over time (Figure 4B) which is comparable to the decrease found for ms^2^C (Figure 4C). For ms^2^C, we observed a slower repair in the absence of AlkB. Intriguingly, ms^4^U loss is independent of AlkB. We concluded that AlkB is not the demethylase of ms^4^U; this opens the way for two hypotheses. The first revolves around a potential, undescribed demethylase or dethiomethylase, which has ms^4^U‐damaged tRNA as substrate. SelU, a dethiogeranylase might be a potential candidate for this reaction^[17]^. From a chemical perspective, a direct dethiomethylation through attack of a nucleophile such as water is also theoretically possible. In both scenarios, ms^4^U would dethiomethylate to uridine, which is again substrate for enzymatic thiolation. The re‐thiolation during the recovery phase can be monitored by analysis of [^34^S] incorporation into original tRNA. Our NAIL‐MS study indeed indicates an increased formation of [^34^S]‐ms^4^U in original tRNA from MMS stressed compared to unstressed bacteria (Figure 4D). This *in vivo* data hints at dethiomethylation of damaged tRNA that results in uridine.

**Figure 4:**
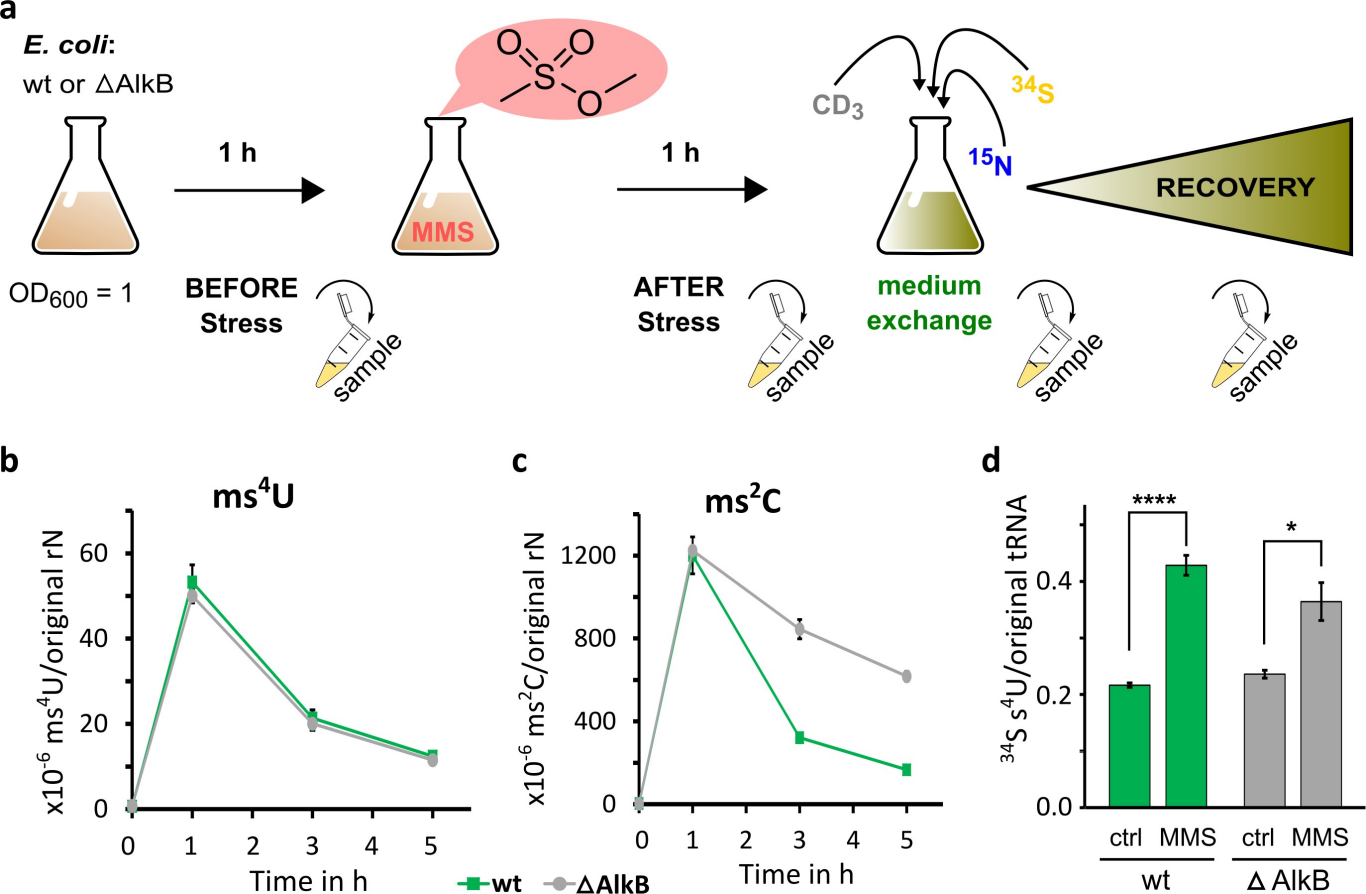
A) Principle of a pulse‐chase NAIL‐MS experiment. The bacteria are grown in unlabeled medium before and after exposure to MMS. After 1 h of exposure to MMS, the medium is replaced with [^15^N]‐, [^34^S]‐ and [CD_3_]methionine‐containing medium. B) Formation and loss during recovery of ms^4^U after exposure to 20 mM MMS in wild‐type (wt, green) and AlkB‐deficient (ΔAlkB, gray) *E. coli*. C) Formation and loss during recovery of ms^2^C after 20 mM MMS exposure in wild‐type (wt, green) and AlkB‐deficient (ΔAlkB, gray) *E. coli*. D) Abundance of [^34^S]ms^4^U after 5 h in control (ctrl) and MMS‐exposed wt and AlkB‐deficient bacteria. All data from 3 biological replicates. Error bars represent standard deviation.

While we cannot exclude the involvement of an unknown dethiomethylase, we tested the possibility of spontaneous ms^4^U dethiomethylation. For this purpose, we simulated potential cellular environments and exposed synthesized ms^4^U as free nucleoside prior to quantitative LC–MS/MS analysis (Figure S2). Dethiomethylation was observed after incubation with dithiothreitol (DTT). No dethiomethylation was observed under acidic/alkaline conditions, in growth medium or in the presence of cysteine or bovine serum albumin (BSA as an example protein).

In summary, we describe the existence of thiomethylated s^4^U in bacterial tRNA. The low abundance of ms^4^U indicates its formation as a lesion through the constantly present electrophile *S*‐adenosylmethionine. During the exposure of bacteria to methylating agents such as MMS, RNA is damaged, and the methylation products of canonical nucleosides (m^1^A, m^7^G, m^3^C, m^3^U and m^6^A) emerge.

In addition, modified nucleosides with a pronounced nucleophilic character, such as s^2^C and s^4^U, become methylated. As evident from Figure 3 (right), s^2^C is more prone to direct methylation than s^4^U. This can be explained by both the chemical reactivity of the S2 in cytidine compared to the S4 in uridine and its location within the tRNA. Due to the exocyclic amine in cytidine, s^2^C has an increased electron density, which improves its nucleophilic character over the S4 in uridine. Furthermore, the uridine S4 is more prone to solvation, which further decreases its nucleophilicity. In addition to the difference in nucleophilicity, s^2^C is exposed and accessible in the anticodon loop of the tRNA, whereas s^4^U is buried in the D‐/T‐loop fold.

Our studies reveal a differential reaction of the cells towards these forms of RNA damage. One class of lesions is repaired through enzymatic demethylation using an oxidative demethylation mechanism. Namely, m^1^A, m^3^C (Figure S3a, b) and ms^2^C (Figure 4C) are substrate to enzymatic demethylation through AlkB. The second class comprises lesions that are lost from the RNA over time, but in an AlkB‐independent manner (ms^4^U and m^6^A). The third class of RNA damage comprises m^7^G, which is not removed from tRNA (Figure S3d).

Overall, the finding of ms^4^U as a natural and stress‐induced lesion in bacterial tRNA confirms the importance of tRNA modifications during stress response.

## Conflict of interest

The authors declare no conflict of interest.

## Supporting information

As a service to our authors and readers, this journal provides supporting information supplied by the authors. Such materials are peer reviewed and may be re‐organized for online delivery, but are not copy‐edited or typeset. Technical support issues arising from supporting information (other than missing files) should be addressed to the authors.

Supplementary
