# Supplementary material for: Synthesis and Metabolic Fate of 4‐Methylthiouridine in Bacterial tRNA
Source: Chembiochem. 2020 Jun 18;21(19):2768–71. doi: 10.1002/cbic.202000272 (PMC7586944; doi:10.1002/cbic.202000272)
Supplement: Supplementary file 1 — Supplementary [file CBIC-21-2768-s001.pdf]

ChemBioChem

Supporting Information

Synthesis and Metabolic Fate of 4-Methylthiouridine in Bacterial tRNA

Christoph Borek[+], Valentin F. Reichle[+], and Stefanie Kellner*

Supporting information

a

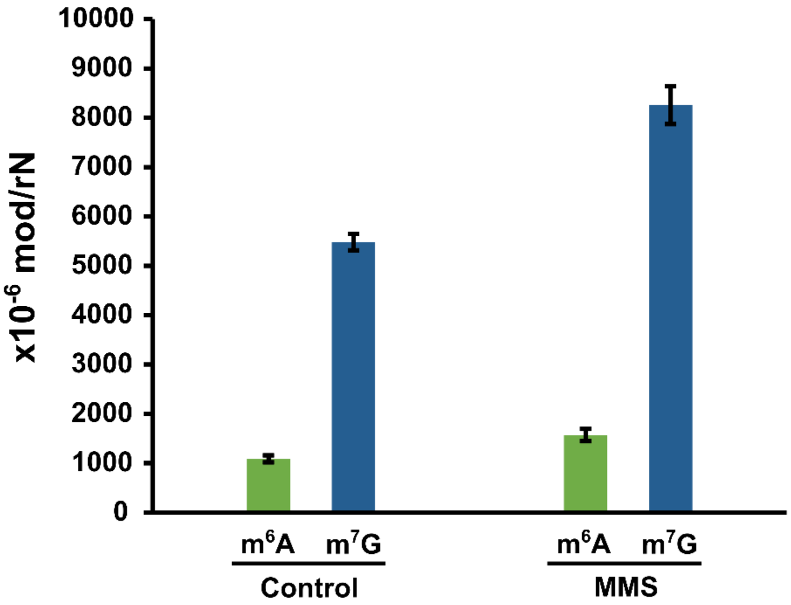

b

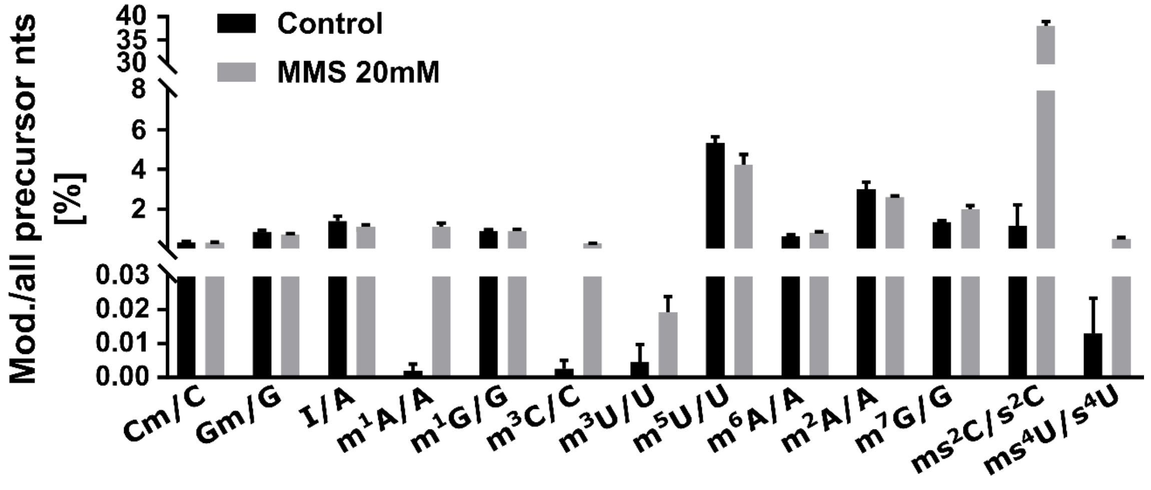

Figure S1: tRNA damage products after 20 mM MMS stress. All experiments are from n=3 biol. replicates and error bars reflect standard deviation. **a** Increase of m^6A (green) and m^7G (blue) after MMS stress in 10^{-6} modifications per ribonucleotide (rN). **b** Percentage of methylated nucleotide in relation to its respective precursor nucleotide (nts).

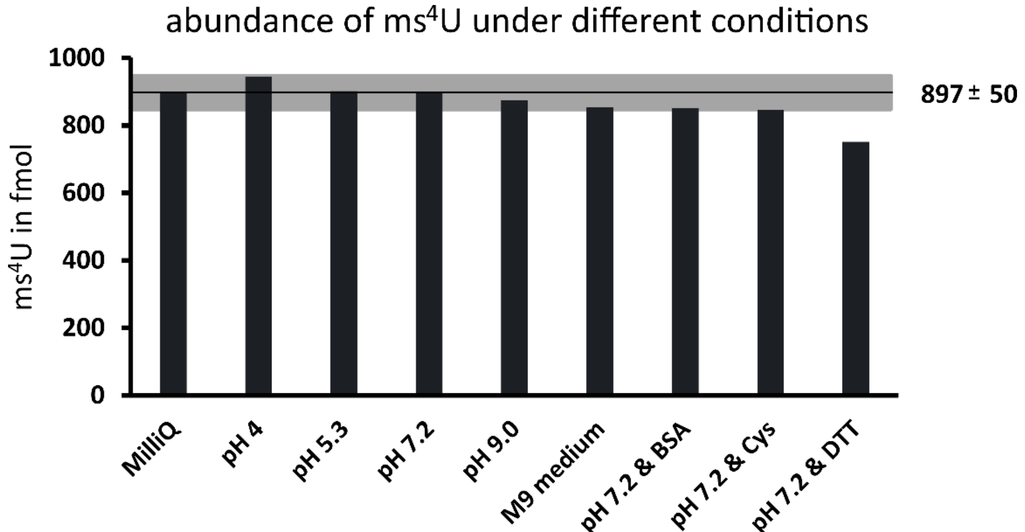

Figure S2: Stability test for ms^{4}U *in vitro*. 900 fmol ms^{4}U were analyzed by LC-MS/MS after 2h at 37°C under different conditions. The grey horizontal bar visualizes a variance of ±50 fmol (±5.6%).

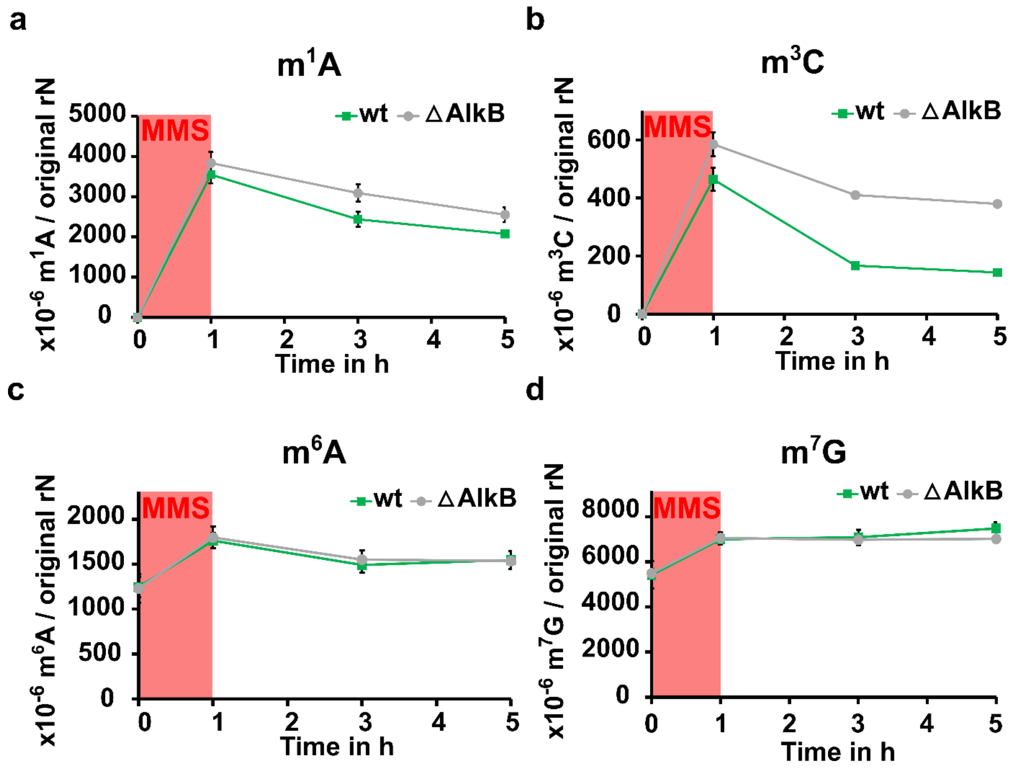

Figure S3: Pulse-chase NAIL-MS study of modified nucleosides not shown in Figure 4. MMS stress (20 mM, 1 h exposure time highlighted in red) in *E. coli* wt (green) and ΔAlkB (grey) strain. The results for m^{1}A (**a**), m^{3}C (**b**), m^{6}A (**c**) and m^{7}G (**d**) are shown. The y-axes scale shows the modification abundance x10^{-6} per original ribonucleotide (rN). All experiments are from n=3 biol. replicates and error bars reflect standard deviation.

Material and Methods

Salts, reagents, and nucleosides

All salts were obtained from Sigma-Aldrich (Munich, Germany) at molecular biology grade unless stated otherwise. The isotopically labeled compounds $^{15}NH_4Cl$ (>98 atom %) and $[D_3]$-L-methionine (98atom %) were obtained from Sigma-Aldrich. Isotopically labelled $^{13}C_6$-glucose (≥99 atom %) and isotopically labeled $Na_2{}^{34}SO_4$ (99.11 atom %) were obtained from Eurisotope (Saarbruecken, Germany). All solutions and buffers were made with water from a Millipore device (Milli-Q, Merck, Darmstadt, Germany). The nucleosides adenosine, cytidine, guanosine, uridine, and N2-methylguanosine (m^2G) were obtained from Sigma-Aldrich. 1-Methyladenosine (m^1A), N3-methylcytidine (m^3C), N6-methyladenosine (m^6A), 7-methylguanosine (m^7G), 5-methyluridine (m^5U), 2'-O-methylcytidine (Cm), 2′-O-methylguanosine (Gm), 1-methylguanosine (m^1G), and 3-methyluridine (m^3U) were obtained from Carbosynth (Newbury, UK). The nucleoside ms^2C was synthesized as described before.[1]

Strains

E. coli strains. The used *E. coli* wild-type strain BW25113 and the isogenic AlkB knockout strain JW2200-KC were purchased from the Keio database[2] All cultures were grown in a shaking incubator at 37 °C at 250 rpm (Orbit=10 mm). Overnight cultures were grown in 5 mL of the differently labeled M9 media used for the respective experiment. The cells were grown starting with an OD_{600} of 1 and grown until reaching stationary phase (OD_{600}~ 4).

M9 media

M9 minimal medium was used with and without the indicated isotopes. Unlabeled M9 was prepared by mixing a 10 × M9 stock solution with glucose, $MgCl_2$, Na_2SO_4, and $CaCl_2$ (as detailed below). For unlabeled 10 × M9 stock solution, Na_2HPO_4 (68 g/L), KH_2PO_4 (30 g/L), NaCl (2.5 g/L), and NH_4Cl (10 g/L) were mixed and autoclaved. For ^{15}N-labeled 10 × M9 stock solution, $^{15}NH_4Cl$ (10 g/L) was used. $MgCl_2$ (0.1 M), $CaCl_2$ (0.1 M), Na_2SO_4 (0.1 M), and 20% (wt%) glucose were prepared by sterile filtration. A 20% (wt%) $^{13}C_6$-labeled glucose solution was prepared for ^{13}C-labeled M9 media. For ^{34}S-labeled M9 media a 0.1 M $Na_2{}^{34}SO_4$ solution was prepared. Final M9 media was prepared by mixing, *e.g.*, 500µL M9 stock solution with 100µL glucose, 100µL $MgCl_2$, 100µL Na_2SO_4, 5µL $CaCl_2$ and water to a final volume of 5 mL. For ^{15}N-labeled cultures, the ^{15}N-10 ×M9 stock solution was used. For ^{13}C-labeled cultures, the 20% (wt%) $^{13}C_6$-labeled glucose solution and for ^{34}S-labeled cultures, the 0.1 M $Na_2{}^{34}SO_4$ solution was used. For CD_3-methylome labeling, 200µL CD_3-methionine (stock 5 g/L) was added to 5 mL of culture volume.

MMS- stress studies *in vitro*

The pipetting scheme for the different *in vitro* sample mixtures is shown in **Table S1**. The buffer solutions were prepared in MilliQ and adjusted with a pH-meter to the respective pH. M9 medium was not pH adjusted but used in the composition as described before. The BSA solution was prepared from a stock solution (c = 10 µg/µl, in PBS) and diluted with MilliQ. DTT and Cysteine were both solved and diluted with MilliQ.

The prepared sample mixtures were incubated in a heating block at 37°C for 2h. To the sample solutions 900 µL MilliQ were added and the mixtures were vortexed (1:10 dilution). Afterwards 18 µL of each sample solution were combined with 2 µL of our SILIS. The LC-MS/MS injection amount was 10 µL, which means a theoretical ms^4U amount of 900 fmol.

Table S1: Pipetting scheme for *in vitro* ms^4U stability test. Abbreviations: Vol. = Volume; Dil. = Dilution; BSA = Bovin serum albumin; Cys = Cysteine; DTT = Dithiothreitol; eq. = Equivalents respective ms^4U.

Sample	Buffer	Vol. ms^4U (0.01 mM)	Vol. Buffer	Add-on	Vol. Add-on	Dil. SILIS
MilliQ	-	10 µL	-	-	-	1:10
pH 4.0	0.1 M Citric Acid	10 µL	90 µL	-	-	1:10
pH 5.3	0.5 mM $NH_4OAc/AcOH$	10 µL	90 µL	-	-	1:10
pH 7.2	0.1 M NH_4HCO_3	10 µL	90 µL	-	-	1:10
pH 9.0	0.1 M $NH_4OAc/NaOH$	10 µL	90 µL	-	-	1:10
M9 medium	0.05 M Na_2HPO_4 0.02 M KH_2PO_4	10 µL	90 µL	-	-	1:10
pH 7.2 & BSA	0.1 M NH_4HCO_3	10 µL	80 µL	0.02 mM BSA	10 µL (2 eq.)	1:10
pH 7.2 & Cys	0.1 M NH_4HCO_3	10 µL	80 µL	0.1 mM Cysteine	10 µL (10 eq.)	1:10
pH 7.2 & DTT	0.1 M NH_4HCO_3	10 µL	80 µL	0.1 mM DTT	10 µL (10 eq.)	1:10

MMS- stress studies *in vivo*

A 5 mL bacterial solution with an OD_{600} of 1 was prepared from an overnight culture. After 60 min growth, 8.5 µL MMS (final conc. 20 mM) were added. As a control, 8.5 µL water was added to a second culture. After 60 min of exposure, the RNA was isolated and total tRNA purified by SEC.

Pulse-chase NAIL-MS experiment

A single colony of *E. coli* BW25113 or *E. coli* JW2200-KC (ΔalkB) was picked and grown in unlabeled M9 medium (5 mL) overnight. From the first overnight culture, a 50 mL culture was prepared in unlabeled M9 medium and grown overnight. From the second overnight culture, 120 mL culture ($OD_{600}$of 1.0) was prepared in unlabeled M9 medium. After 60 min growth, the first aliquot (7 mL) was taken for RNA isolation. The remaining culture was split into two flasks of 56.5 mL each. One was exposed to MMS (95.7µL, 20 mM final concentration) the other to water (MOCK) and inverted before both cultures were cultivated for 60 min. An aliquot (7 mL) was drawn from each culture, and the RNA of the aliquot was isolated. The remaining bacteria were centrifuged (1200 ×g, 5 min), and the MMS/MOCK-containing supernatants were discarded. The bacteria pellets were

washed with ^{15}N, ^{34}S and CD_3-methionine labeled M9 medium (5 mL), and each bacterial pellet was suspended in fresh $^{15}N/^{34}S/CD_3$ M9 medium (50 mL). The bacteria were allowed to grow and recover from the MMS/MOCK treatment. Seven mL of each bacterial culture were harvested after 2 and after 4 h. The RNA was isolated and the tRNA purified by SEC.

SILIS preparation

A single colony of *E. coli* BW25113 was picked and grown in $^{15}N/^{13}C$ labeled M9 medium (10 mL) overnight. The overnight culture was diluted with 90 mL freshly prepared $^{15}N/^{13}C$ labeled M9 medium and it was incubated till the OD_{600} reached 1.0. The culture was then exposed to MMS (170 µL, 20 mM final concentration) and after 60 min the cells were harvested and the RNA was isolated and digested as described in the protocols below.

Cell lysis and tRNA purification

The bacteria culture was centrifuged at 1200 ×g for 5 min. The supernatant was discarded and the cell pellet was resuspended in 1 mL TRI reagent (Sigma-Aldrich) per 5 mL bacteria culture. The total RNA was isolated according to the supplier's manual. tRNA was purified by size exclusion chromatography (SEC) according to published procedures.[3] The tRNA was resuspended in water (30µL).

tRNA digestion for mass spectrometry

tRNA (100 ng) in aqueous digestion mix (30µL) was digested to single nucleosides by using 0.2 U alkaline phosphatase, 0.02 U phosphodiesterase I (VWR, Radnor, Pennsylvania, USA), and 0.2 U benzonase in Tris (pH 8,5 mM) and $MgCl_2$ (1 mM) containing buffer. Furthermore, 0.5 µg tetrahydrouridine (Merck, Darmstadt, Germany), 1 µM butylated hydroxytoluene, and 0.1 µg pentostatin were added to avoid deamination and oxidation of the nucleosides.[3] The mixture was incubated for 2 h at 37 °C and 1/10 Vol. of SILIS (stable isotope labeled internal standard) was added to each sample before analysis by QQQ mass spectrometry.

QQQ mass spectrometry

For quantitative mass spectrometry an Agilent 1290 Infinity II equipped with a diode-array detector (DAD) combined with an Agilent Technologies G6470A Triple Quad system and electrospray ionization (ESI-MS, Agilent Jetstream) was used. Operating parameters: positive-ion mode, skimmer voltage of 15 V, cell accelerator voltage of 5 V, N2 gas temperature of 230 °C and N2 gasflow of 6 L/min, sheath gas (N2) temperature of 400 °C with a flow of 12 L/min, capillary voltage of 2500 V, nozzle voltage of 0 V, and nebulizer at 40 psi. The instrument was operated in dynamic MRM mode. We used a flow rate of 0.35 mL/min in combination with a binary mobile phase of 5 mM NH_4OAc aqueous buffer A, brought to pH 5.6 with glacial acetic acid (65µL in 1 L), and an organic buffer B of pure acetonitrile (Roth, LC-MS grade, purity≥.99.95). The *in vitro* experiments, the coinjects and the metabolic CD_3 labeling digest seperation was performed on a Synergi Fusion-RP column (Phenomenex®, Torrance, CA, USA; Synergi® 2.5 µm Fusion-RP 100Å, 150 x 2.0 mm) at 35 °C. The gradient started with 100% A for one minute, increased to 10% B by 5 min, and to 40% B by 7 min. The column was flushed with 40% B for 1 min and returned to starting conditions to 100% A

by 8.5 min followed by re-equilibration at 100% A for 2.5 additional minutes. For the pulse-chase experiment and the unlabeled *in vivo* MMS stress study (Figure 3), the digests were separated on a Core-Shell Technology column (Phenomenex, Torrance, CA, USA; Kinetex 1.7µm EVO C18, 100 Å, 150 × 2.1 mm) at 35 °C. The gradient started at 100% solvent A, followed by an increase to 10% over 10 min. From 10 to 15 min, solvent B was increased to 45% and was maintained for 3 min before returning to 10% solvent A and a 3 min re-equilibration period.

Table S2: MRM parameters for co-injection, CD_3-metabolic labeling and MMS *in vitro* experiments.

Comp. group	Comp. Name	Precursor Ion (m/z)	Product Ion (m/z)	Ret Time (min)	Fragmentor (V)	CE (V)	Cell Acc (V)	Polarity
not labeled	C	244	112	2	200	40	5	Positive
	Cm	258	112	3.8	180	9	5	Positive
	m^3C	258	126	1.7	88	14	5	Positive
	m^3U	259	127	4.4	75	9	5	Positive
	m^5U	259	127	4.1	145	10	5	Positive
	ms^2C	274	142	4.5	85	13	5	Positive
	ms^4U	275	143	6.3	70	12	5	Positive
	s^2C	260	128	3.2	85	13	5	Positive
	s^4U	261	129	4.6	75	17	5	Positive
	U	245	113	2.8	95	5	5	Positive
SILIS	C SILIS	256	119	2	200	40	5	Positive
	Cm SILIS	271	119	3.8	180	9	5	Positive
	m^3C SILIS	270	133	1.7	88	14	5	Positive
	m^3U SILIS	271	134	4.4	75	9	5	Positive
	m^5U SILIS	271	134	4.1	145	10	5	Positive
	ms^2C SILIS	286	149	4.5	85	13	5	Positive
	ms^4U SILIS	286	149	6.3	70	12	5	Positive
	s^2C SILIS	272	135	3.2	85	13	5	Positive
	s^4U SILIS	272	135	4.6	75	17	5	Positive
	U SILIS	256	119	2.8	95	5	5	Positive
^{13}C	ms^4U ^{13}C	284	147	6.3	70	12	5	Positive
^{15}N	ms^4U ^{15}N	277	145	6.3	70	12	5	Positive
^{34}S	ms^4U ^{34}S	277	145	6.3	70	12	5	Positive
CD_3 - labeled	ms^4U CD_3	278	146	6.3	70	12	5	Positive
	Cm CD_3	261	112	3.8	180	9	5	Positive
	m^5U CD_3	262	130	4.1	145	10	5	Positive

Table S3: MRM parameters for pulse-chase experiments and unlabeled 20 mM MMS *in vivo* stress experiment (Figure 3).

Comp. group	Comp. Name	Ret. Time (min)	Precursor Ion (m/z)	Product Ion (m/z)	Fragmentor (V)	CE (V)	Cell Acc (V)	Polarity
not labeled (original)	A	5.611	268	136	200	40	5	Positive
	C	1.73	244	112	200	40	5	Positive
	Cm	3.347	258	112	180	9	5	Positive
	G	3.718	284	152	200	40	5	Positive
	Gm	4.78	298	152	100	9	5	Positive
	I	3.272	269	137	100	9	5	Positive
	m^1A	1.745	282	150	110	21	5	Positive
	m^1G	4.85	298	166	100	15	5	Positive
	m^3C	1.752	258	126	88	14	5	Positive
	m^3U	4.7	259	127	75	9	5	Positive
	m^5U	3.61	259	127	145	10	5	Positive
	m^6A	7.85	282	150	120	20	5	Positive
	m^7G	2.7	298	166	105	14	5	Positive
	ms^2C	5.533	274	142	85	13	5	Positive
	ms^4U	7.34	275	143	75	17	5	Positive
	s^2C	2.288	260	128	85	13	5	Positive
	s^4U	4.337	261	129	75	17	5	Positive
	U	1.876	245	113	95	5	5	Positive
$^{34}S/^{15}N/CD_3$ labeled (new)	A ^{15}N	5.611	273	141	200	40	5	Positive
	C ^{15}N	1.73	247	115	200	40	5	Positive
	Cm $^{15}N\_CD_3$	3.347	264	115	180	9	5	Positive
	G ^{15}N	3.718	289	157	200	40	5	Positive
	Gm $^{15}N\_CD_3$	4.78	306	157	100	9	5	Positive
	I ^{15}N	3.272	273	141	100	9	5	Positive
	m^1A $^{15}N\_CD_3$	1.745	290	158	110	21	5	Positive
	m^3C $^{15}N\_CD_3$	1.752	264	132	88	14	5	Positive
	m^3U $^{15}N\_CD_3$	4.7	264	132	75	9	5	Positive
	m^5U $^{15}N\_CD_3$	3.61	264	132	145	10	5	Positive
	m^6A $^{15}N\_CD_3$	7.85	290	158	120	20	5	Positive
	m^1G $^{15}N\_CD_3$	4.85	306	174	100	15	5	Positive
	m^7G $^{15}N\_CD_3$	2.7	306	174	105	14	5	Positive
	ms^2C $^{15}N\_CD_3\_^{34}S$	5.533	282	150	85	13	5	Positive
	ms^4U $^{15}N\_CD_3\_^{34}S$	7.34	282	150	75	17	5	Positive
	s^2C $^{15}N\_^{34}S$	2.288	265	133	85	13	5	Positive

	s^4U ^{15}N_^{34}S	4.337	265	133	75	17	5	Positive
	U ^{15}N	1.876	247	115	95	5	5	Positive
**Stable Isotope Labeled Internal Standard (SILIS)**	A SILIS	5.611	283	146	200	40	5	Positive
	C SILIS	1.73	256	119	200	40	5	Positive
	Cm SILIS	3.347	271	119	180	9	5	Positive
	G SILIS	3.718	299	162	200	40	5	Positive
	Gm SILIS	4.78	314	162	100	9	5	Positive
	I SILIS	3.272	283	146	100	9	5	Positive
	m^1A SILIS	1.745	297	160	110	21	5	Positive
	m^1G SILIS	4.85	314	177	100	15	5	Positive
	m^3C SILIS	1.752	270	133	88	14	5	Positive
	m^3U SILIS	4.7	271	134	75	9	5	Positive
	m^5U SILIS	3.61	271	134	145	10	5	Positive
	m^6A SILIS	7.85	298	161	120	20	5	Positive
	m^7G SILIS	2.7	314	177	105	14	5	Positive
	ms^2C SILIS	5.533	286	149	85	13	5	Positive
	ms^4U SILIS	7.34	286	149	75	17	5	Positive
	s^2C SILIS	2.288	272	135	85	13	5	Positive
	s^4U SILIS	4.337	272	135	75	17	5	Positive
	U SILIS	1.876	256	119	95	5	5	Positive
**^{34}S labeled (re-thiolation)**	s^2C ^{34}S	2.288	262	130	85	13	5	Positive
	s^4U ^{15}N or ^{34}S	4.337	263	131	75	17	5	Positive

Calibrations and data analysis

For calibration, synthetic nucleosides were weighed and dissolved in water to a stock concentration of 1–10 mM. In contrast to the ms^4U standard, due to an unknown content of water and salts in the synthesized ms^2C standard, the concentration could not be determined through weighing. Therefore, the concentration of the stock solution was determined by comparison to s^2C containing isoacceptors after MMS exposure.[1] The calibration solutions range from 0.3 to 500 pmol for each canonical nucleoside and from 0.3 to 500 fmol for each modified nucleoside and were spiked with 10% SILIS. The sample data were analyzed by the Quantitative and Qualitative MassHunter Software from Agilent. The areas of the MRM signals were integrated for each modification and their isotope derivatives. The absolute amounts of the modifications were referenced to the absolute amounts of the respective canonical. In the case of the pulse-chase experiment, the different isotopomers were referenced to their respective labeled canonicals, so that original modifications were referenced to original canonicals and new

modifications were referenced to new canonicals. See Eqs. (1) and (2) for s^4U as an example in **Table S4.**

Table S4: Equations for absolute quantification calculations in the pulse-chase experiment. The ms^4U precursor molecule s^4U was chosen here as an example modification.

	s^4U (fmol)	U (fmol)	Normalization
(1) original	$\frac{\text{area s4U (unlabeled)}}{\text{rRFN } s4U \times \text{area s4U (SILIS)}}$	$\frac{\text{area U (unlabeled)}}{\text{rRFN U} \times \text{area U (SILIS)}}$	$\frac{\text{s4U (original)}}{\text{U (original)}}$
(2) new	$\frac{\text{area s4U (34S, 15N, CD3)}}{\text{rRFN s4U} \times \text{area s4U (SILIS)}}$	$\frac{\text{area U (15N)}}{\text{rRFN U} \times \text{area U (SILIS)}}$	$\frac{\text{s4U (new)}}{\text{U (new)}}$

Synthetic access to 4-methylthiouridine

All chemicals were purchased from AlfaAesar, Acros, or Sigma Aldrich in standard purity and used without further purification if not stated otherwise. NMR-Spectra were measured on a *Bruker* Fourier 300 or *Bruker* Avance III HD 300. The chemical shift δ is given ppm, coupling constants J in Hz. As internal standard remaining Protons of the deuterated solvents and their carbons were used with the following shifts:

Solvent	δ (ppm) ^1H	δ (ppm) ^{13}C
$CDCl_3$	7.260	77.160
d^6-DMSO	2.500	39.520
MeOD	4.870	49.000
D_2O	4.790	

The multiplicities are given with the following abbreviations: s = singlet, s(br) = broad singlet, d = doublet, t = triplet, q = quartet, m = multiplet as well as combinations according to the scheme: dt = doublet from triplet. The NMR signals were assigned via DEPT90, DEPT135, 2D correlation spectra (COSY, HSQC, HMBC, NOESY).

TLC were performed on precoated sheets ALUGRAM® Xtra SIL G/UV_{254} by *Macherey-Nagel*. Fluorescence quenching was observed at 254 nm. Composition of mobile phases (eluents) are given in volume fractions (v/v), as standard staining reagent for the sugar compounds anisaldehyde/sulfuric acid was used. Column chromatography was was carried out with columns whose dimensions were adapted to the respective separation problem packed with Silica 60 (0,06 - 0,2 mm) from *Roth*.

Syntheses

2,3,5-Tri-*O*-acetyl uridine (**1**)

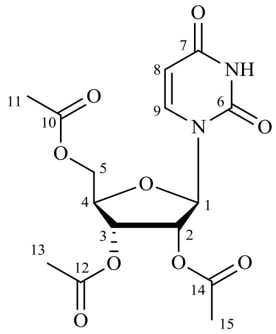

To a suspension of 2.15 g Uridine (8.8 mmol, 1 eq.) in 15 ml of acetic anhydride was added a catalytical amount of Iodine. The mixture was stirred at room temperature until all starting material was consumed (about 30 min) and further 15 minutes to complete the reaction (TLC control). Then aqueous sodium thiosulfate solution was added until the solution was completely decolorized. A bath with cold water was applied and three drops of concentrated sulfuric acid was added to decompose the excess of acetic anhydride. The mixture was stirred vigorously for 20 minutes until no further heat development indicated the completion of the reaction. The reaction mixture was portioned between water and chloroform, the water phase was extracted two times with chloroform, and the combined organic layers were thoroughly washed with water and finally with concentrated sodium hydrogen carbonate solution. After drying with anhydrous sodium sulfate, the solvent was removed and the remaining resin was dried in high vacuum to give the peracetylated uridine as a colourless foam.

yield: 3.00 g (7.1 mmol, 92 %)

appearence: Colorless foam

R_f : 0.07 (cyclohexane : ethylic acetat [1:1]) / 0.4 (cyclohexane : ethylic acetat [1:4])

^{1}H-NMR: (300 MHz CDCl3) δ 9.95 (s, 1H, -N***H***), 7.37 (d, J = 8.1 Hz, 1H, H9), 5.99 (d, J = 4.6 Hz, 1H, H1), 5.74 (d, J = 8.2 Hz, 1H, H8), 5.35 – 5.24 (m, 2H, H2/H3), 4.29 (s, 3H, H4/H5/H5´), [2.08 (s, 3H), 2.06 (s, 3H), 2.04 (s, 3H) 9x H11+13+15] ppm.

^{13}C-NMR: 13C NMR (75 MHz, CDCl3) δ (170.22, 169.66 C10/12/14), 163.26 (C7), 150.45 (C6), 139.50 (C9), 103.35 (C8), 87.43 (C1), 79.83 (C4), (72.67, 70.15 C2/3), 63.17, 20.73 (C5), (20.47, 20.36 C11/13/15) ppm.

2,3,5-Tri-*O*-acetyl thiouridine (**2**)

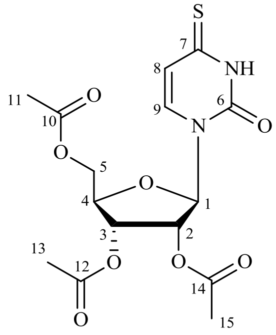

2.00 g peracetyleted uridine (5.4 mmol, 1 eq.) and 2.40 g phosphorous pentasulfide (10.8 mmol, 2 eq.) were dissolved in 40 ml of pyridine and heated to reflux. After 30 min 0.1 ml of water was added und the mixture was refluxed for further 3.5 h. Upon cooling, excessing P_2S_5 participated as yellow solid and was removed by filtration. The filtered solids were washed with pyridin two times and the solvent was removed under reduced pressure. To the residue carefully water was added and stirred, until no further H_2S evolution was observed. During this process, the crude product participated as yellow solid which was separated by filtration, dissolved in chloroform and dried with anhydrous sodium sulfate. The amber glass like residue, which form upon solvent removal was dissolved in methanol and subjected to flash column chromatography (very short column, about 10 cm to remove any remaining polar components) with an eluent gradient cyclohexane/ ethylic acetate 1:1 to 1:4. nochmal schauen

yield: 3.00 g (7.1 mmol, 92 %)

appearence: Colorless foam

R_f : 0.33 (cyclohexane : ethylic acetat [1:1]) / 0.7 (cyclohexane : ethylic acetat [1:4])

^{1}H-NMR: (300 MHz CDCl3) δ 9.95 (s, 1H, -N***H***), 7.37 (d, J = 8.1 Hz, 1H, H9), 5.99 (d, J = 4.6 Hz, 1H, H1), 5.74 (d, J = 8.2 Hz, 1H, H8), 5.35 – 5.24 (m, 2H, H2/H3), 4.29 (s, 3H, H4/H5/H5´), [2.08 (s, 3H), 2.06 (s, 3H), 2.04 (s, 3H) 9x H11+13+15] ppm.

^{13}C-NMR: 13C NMR (75 MHz, CDCl3) δ (170.22, 169.66 C10/12/14), 163.26 (C7), 150.45 (C6), 139.50 (C9), 103.35 (C8), 87.43 (C1), 79.83 (C4), (72.67, 70.15 C2/3), 63.17, 20.73 (C5), (20.47, 20.36 C11/13/15) ppm.

Thiouridine (**3**)

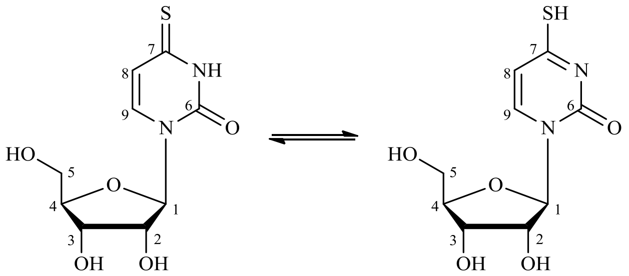

2.04 g (5.2 mmol) peracetylated thiouridine (**2**) were dissolved in 3 ml methanol and mixed with 30 ml of concentrated NH_4OH and refluxed for 2 h until TLC indicated completion of the conversion. The mixture was then neutralized with diluted acetic acid (25 % in water) to approx. pH 6 and the solvents were evaporated under reduced pressure by co-evaporating with ethanol several times to give an orange oil, which can be directly used for the next step. However, a small amount was dissolved in hot isopropylic alcohol, which was mixed with pentane after cooling, which lead to participation of the product. After filtration and drying in high vacuum, the product is highly hygroscopic and electrostatically chargable. Furthermore it undergoes tautomery very fast (on the timescale of NMR-characterization). A reliable assignment to one of the two tautomeric forms is not possible on the basis of the available spectra alone.

**Yield (crude):**	1.22 g (4.7 mmol, 90 %)
**appearence:**	Orange resin as crude product, pale yellow solid after recrystallization
**R_f :**	Not measured
**^{1}H-NMR (of crude product):**	1H NMR (300 MHz, DMSO-d6) δ 7.83 (d, J = 7.6 Hz, 1H, H9), 6.30 (d, J = 7.5 Hz, 1H, H8), 5.73 (d, J = 4.7 Hz, 1H, H1), 4.03 (t, J = 4.8 Hz, 1H, H2), 3.97 (t, J = 4.7 Hz, 1H, H3), 3.91 – 3.82 (m, 1H, H4), 3.65 (dd, J = 12.1, 3.0 Hz, 1H, H5), 3.56 (dd, J = 12.1, 3.2 Hz, 1H, H5´) ppm. Of note, that measured triplets for H3 and H4 in theory are dd, but splitting was not resolved by the used NMR spectrometer.
**^{13}C-NMR (of crude product):**	13C NMR (75 MHz, DMSO) δ 190.35 (C7), 148.13 (C6), 135.95 (C9), 112.64 (C8), 88.57 (C1), 84.97 (C4), 74.00 (C2), 69.57 (C3), 60.53 (C5) ppm

4-Methylsulfanyl-1 β-*D*-ribofuranosyl-1*H*-pyrimidin-2-one MS4U (**4**)

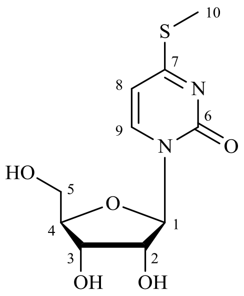

To 1.22 g (4,7 mmol, 1 eq.) Thiouridine (**3**) in 25 ml 50 % ethanol 1.33 g methyl iodide (9.4 mmol, 584 µl, 2 eq.) and 4.7 ml 1 N NaOH were added and the mixture stirred at room temperature for 1 h. Then, the mixture was neutralized with diluted acetic acid (25 % in water) to approx. pH 6. After a short column (flash column chromatography, 10 cm, eluent chloroform/isopropanol 9:1 → pure isopropanol) the product was obtained as highly hygroscopic yellow foam.

yield: 915 mg (3.4 mmol, 71 %)

appearence: Highly hygroscopic yellow foam

R_f : 0.66 (isopropanol)

^{1}H-NMR: (300 MHz, Methanol-d4) δ 8.35 (d, J = 7.2 Hz, 1H, H9), 6.55 (d, J = 7.2 Hz, 1H, H8), 5.92 (d, J = 2.2 Hz, 1H, H1), 4.87 (s, 7H, H2), 4.24 – 4.19 (m, 2H, H2, H3), 4.13 (ddd, J = 6.4, 2.8 Hz, 1H, H4), 3.97 (dd, J = 12.4, 2.6 Hz, 1H, H5), 3.83 (dd, J = 12.4, 3.0 Hz, 1H, H5´), 2.57 (s, 3H, 3 x H10) ppm.

^{13}C-NMR: (75 MHz, MeOD) δ 180.58 (C7), 156.39 (C6), 142.05 (C9), 105.05 (C8), 92.67 (C1), 85.98 (C4), 76.53 (C2), 70.23 (C3), 61.44 (C5), 13.00 (C10) ppm.

NMR-spectra

Of compound (**1**) 2,3,5-Tri-*O*-acetyl uridine

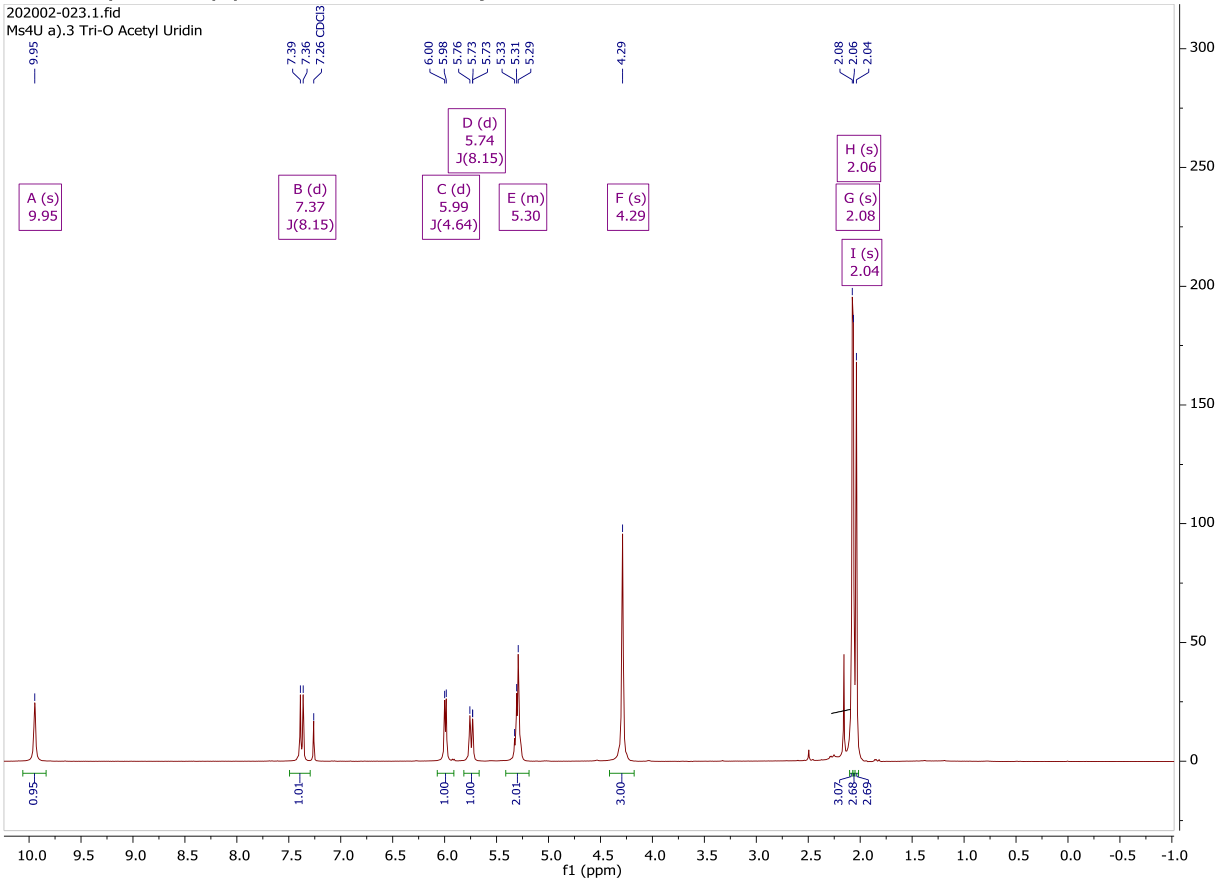

*Figure S4 ^{1}H-NMR spectrum of (**1**)*

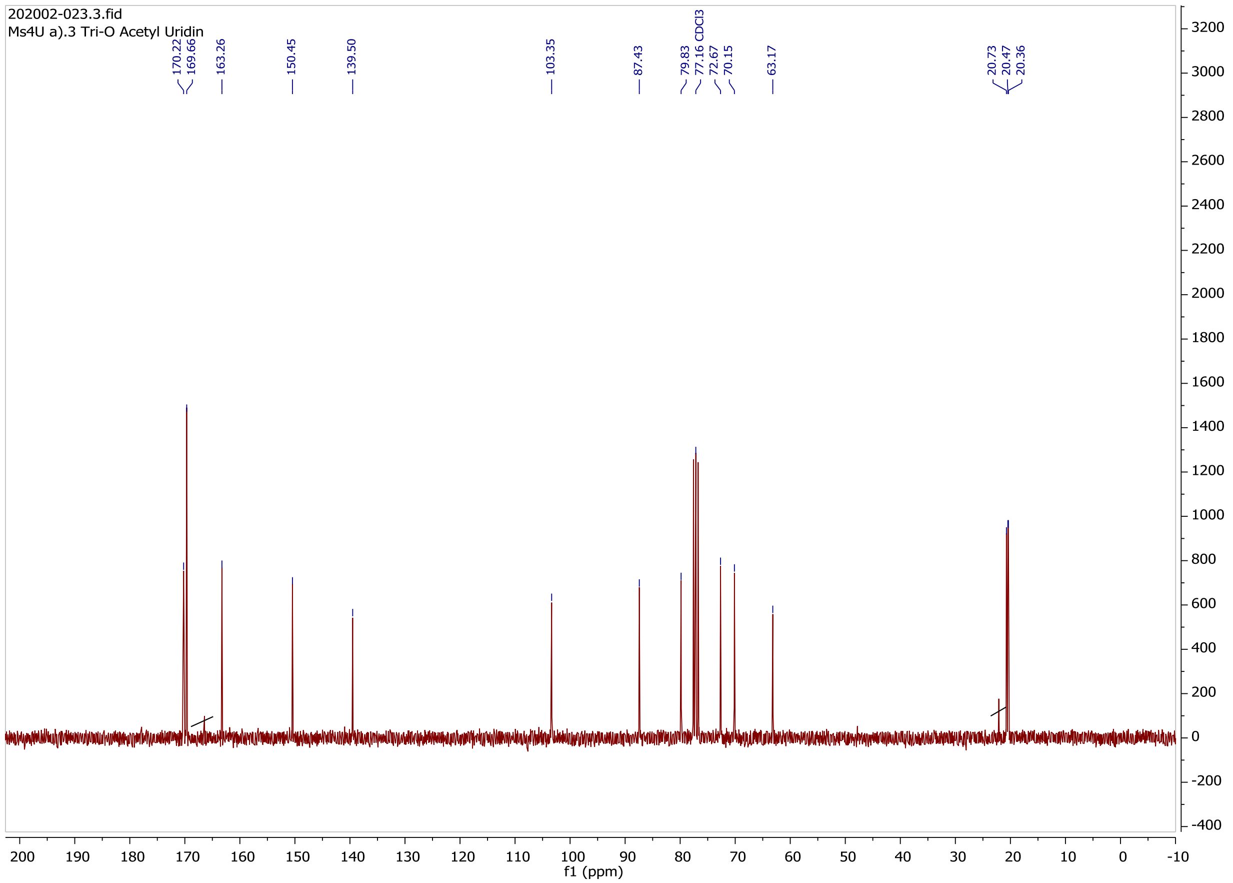

*Figure S5 ^{13}C spectrum of (**1**)*

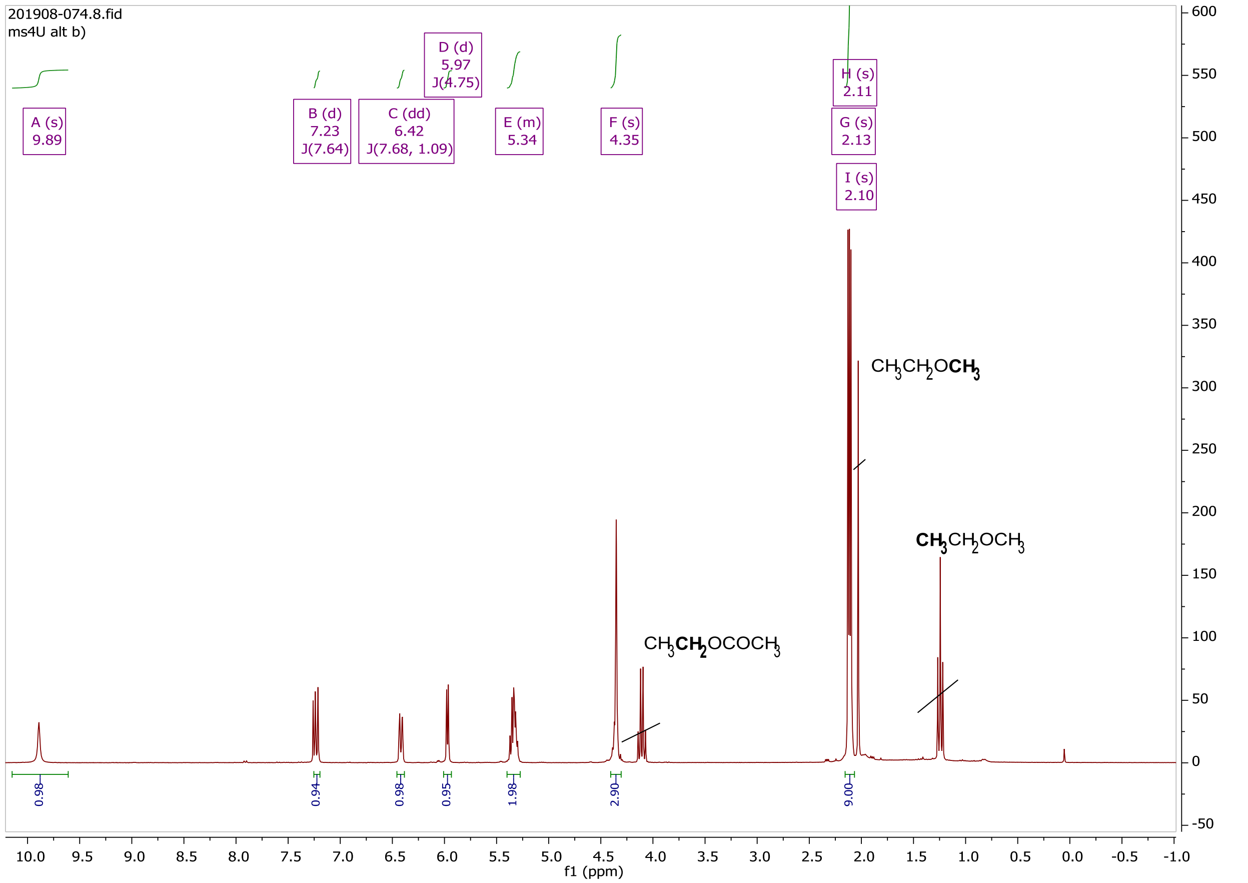

Figure S6 ^{1}H-NMR spectrum of (2)

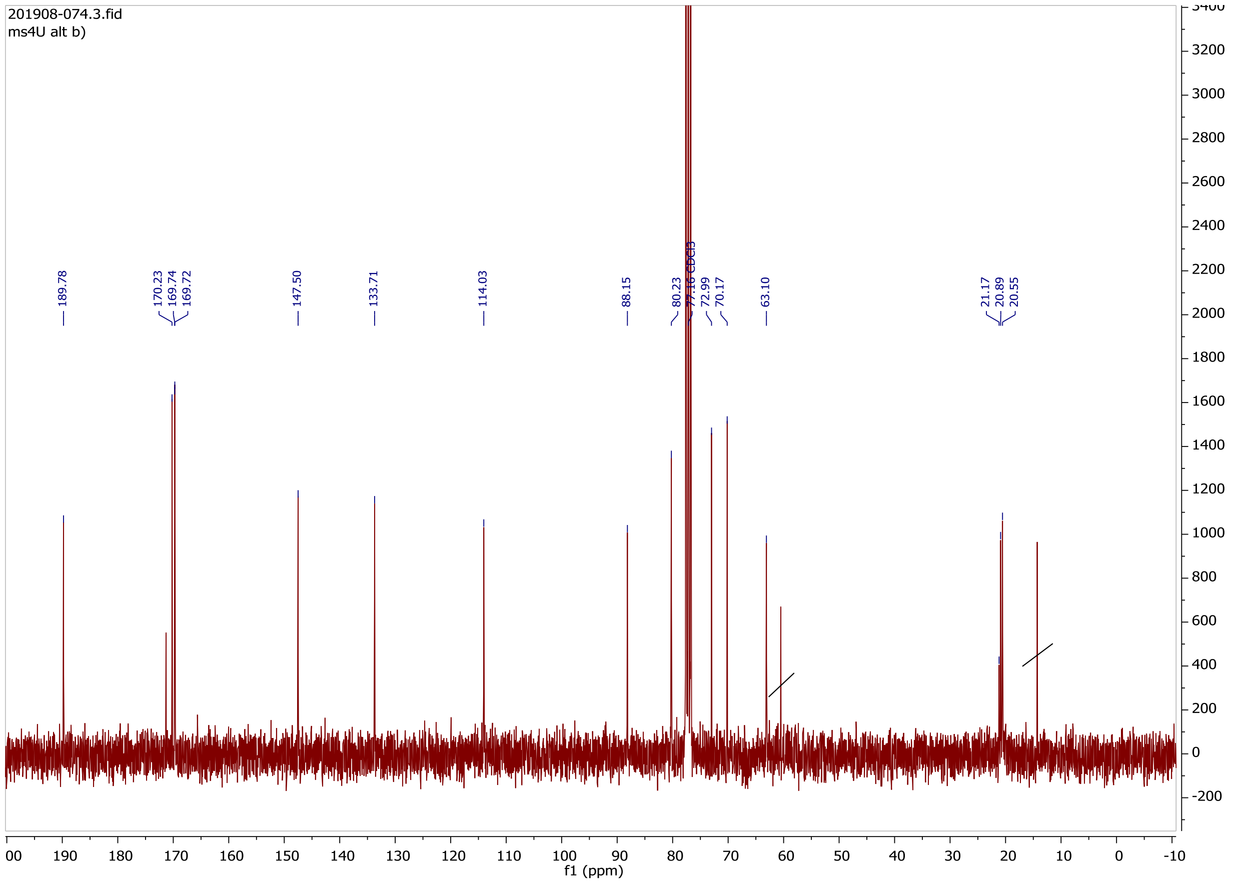

Figure S7 ^{13}C spectrum of (2)

Of compound (**3**) Thiouridine

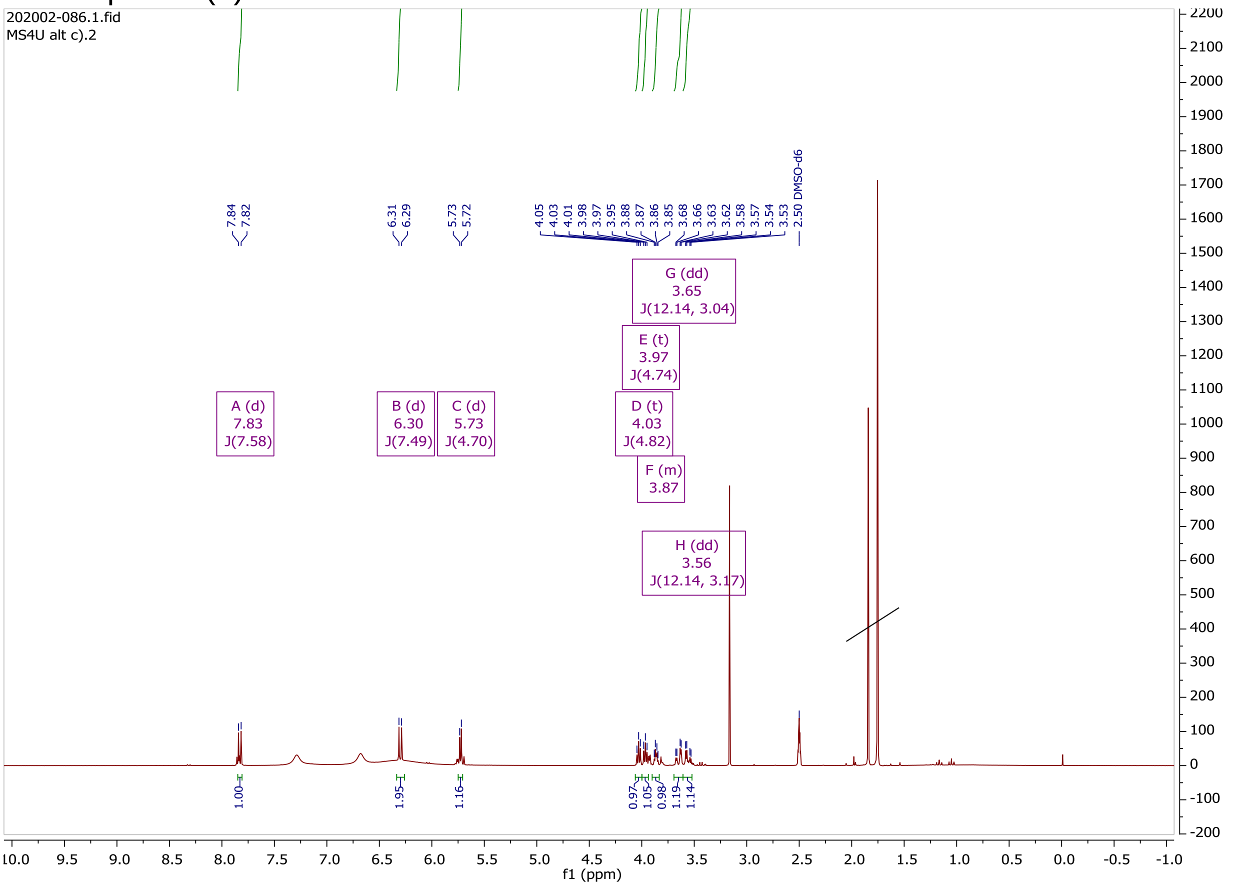

*Figure S8 ^{1}H-NMR spectrum of (**3**)*

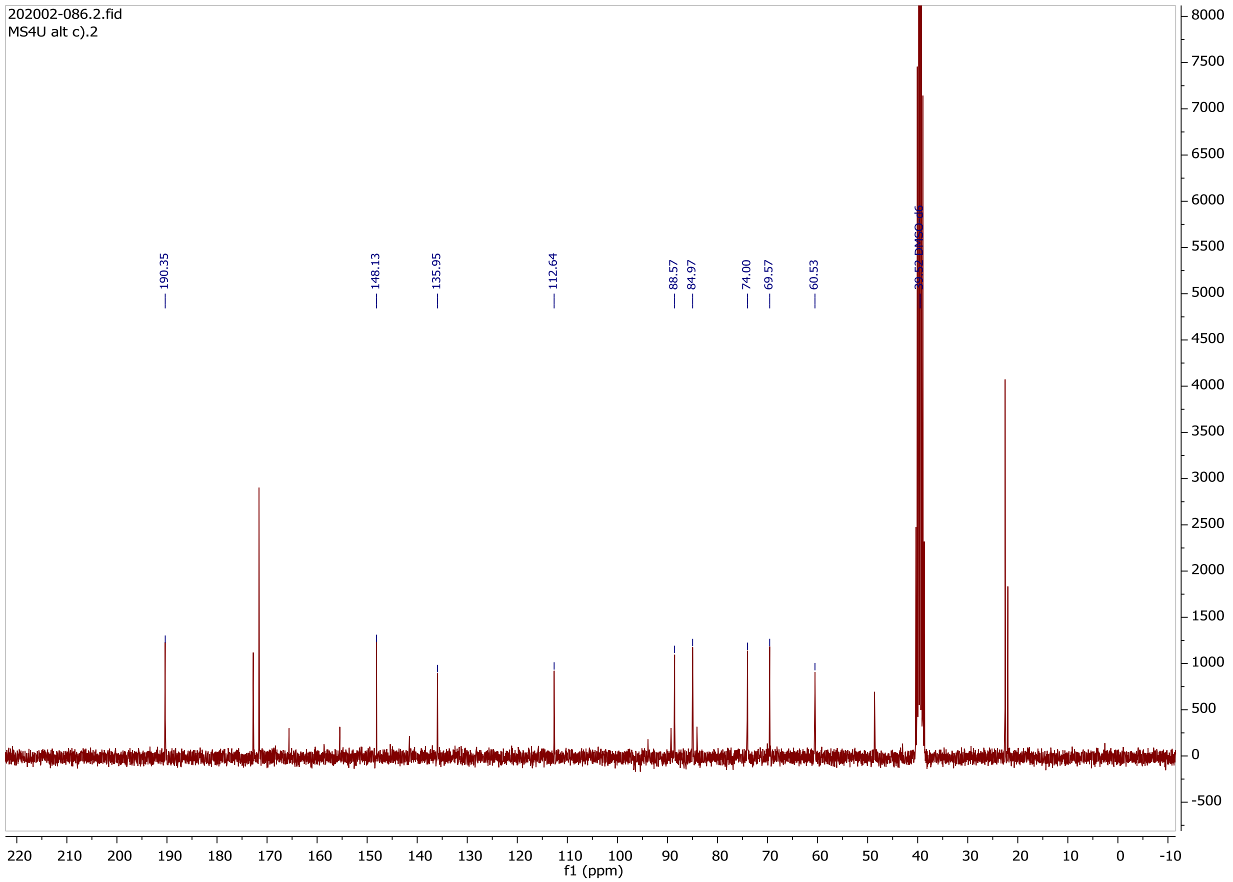

Figure S9 ^{13}*C spectrum of (**3**)*

Of compound (**4**) ms^4U

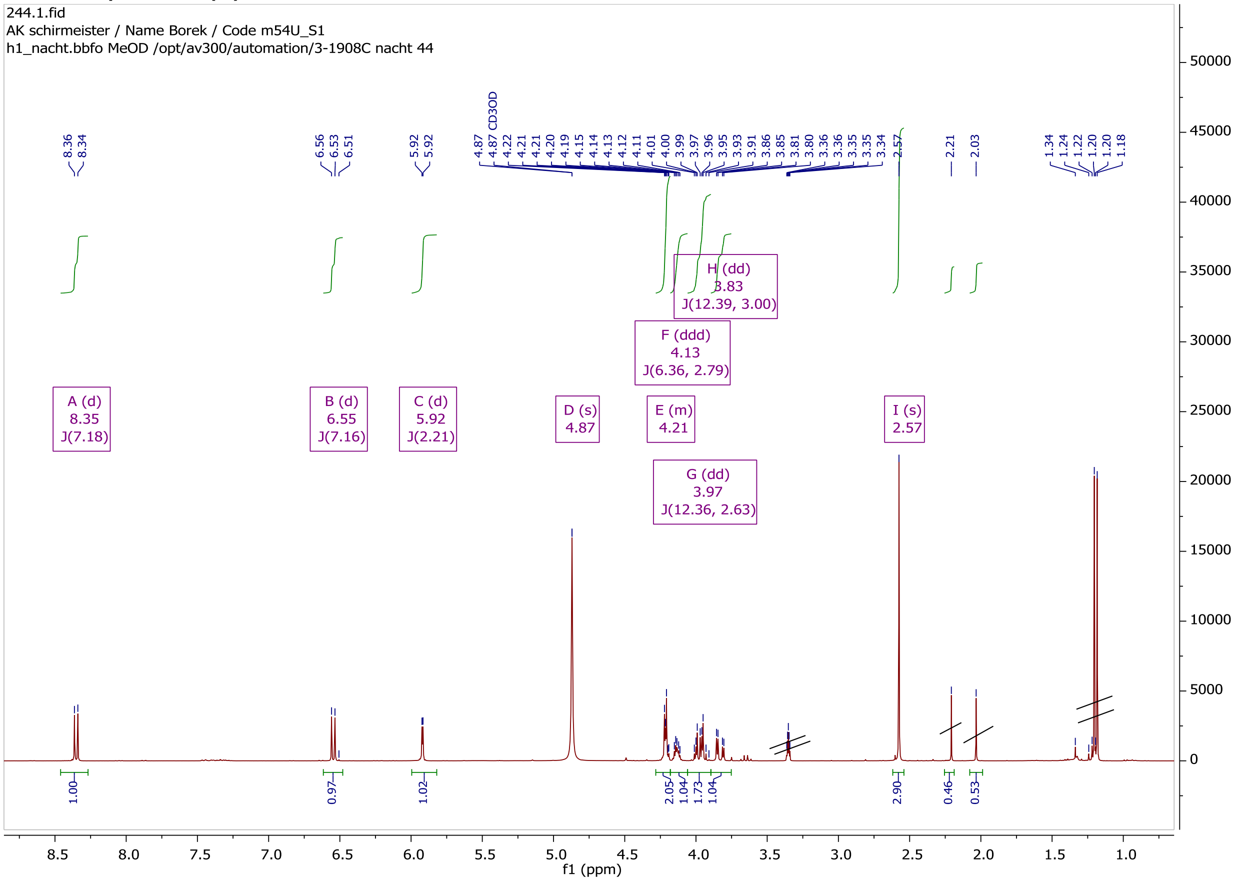

*Figure S10 ^{1}H-NMR spectrum of (**4**)*

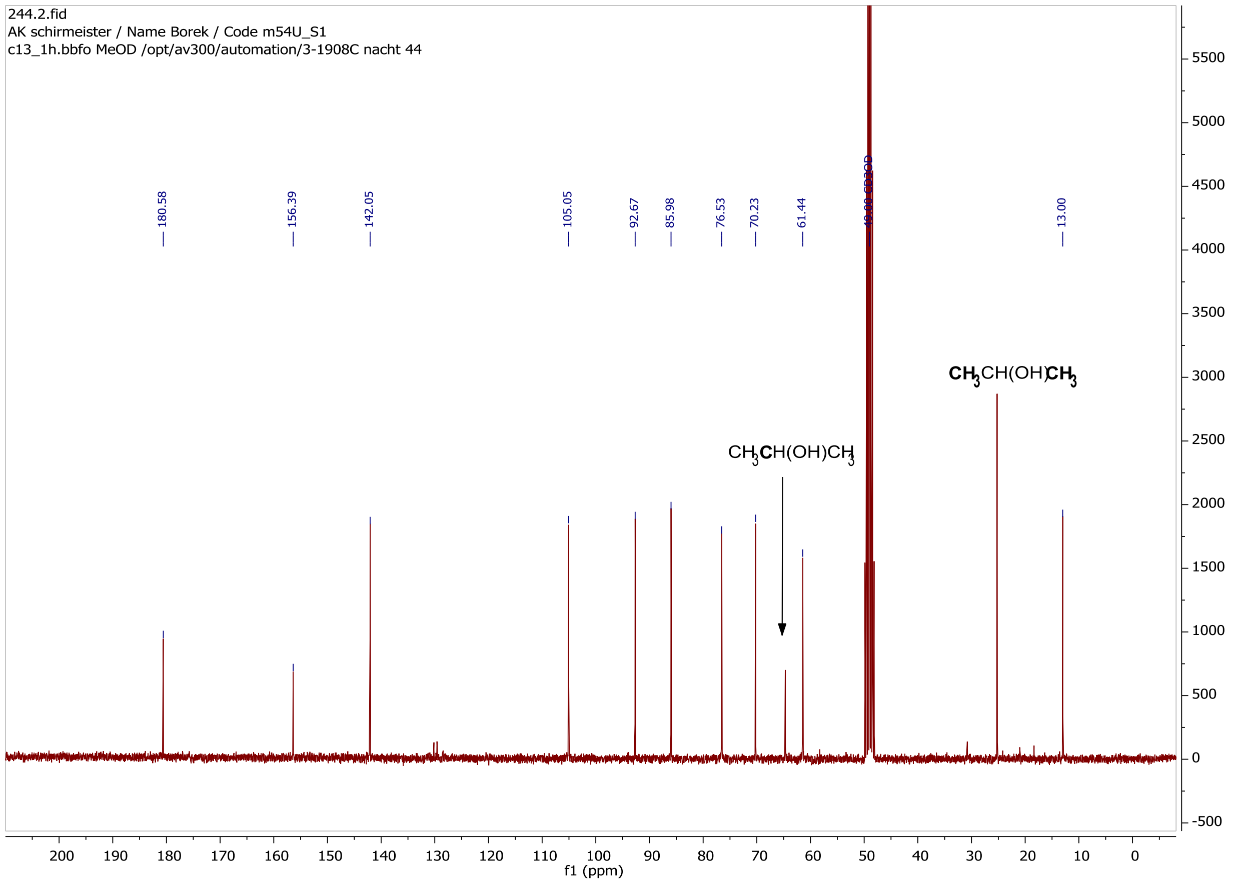

*Figure S11 ^{13}C spectrum of (**4**)*

[1] V. F. Reichle, D. P. Petrov, V. Weber, K. Jung, S. Kellner, *Nature communications* **2019**, *10*, 5600.

[2] T. Baba, T. Ara, M. Hasegawa, Y. Takai, Y. Okumura, M. Baba, K. A. Datsenko, M. Tomita, B. L. Wanner, H. Mori, *Molecular systems biology* **2006**, *2*, 2006.0008.

[3] V. F. Reichle, V. Weber, S. Kellner, *Chembiochem : a European journal of chemical biology* **2018**, *19*, 2575-2583.
